# Simufilam suppresses overactive mTOR and restores its sensitivity to insulin in Alzheimer’s disease patient lymphocytes

**DOI:** 10.3389/fragi.2023.1175601

**Published:** 2023-06-29

**Authors:** Hoau-Yan Wang, Zhe Pei, Kuo-Chieh Lee, Boris Nikolov, Tamara Doehner, John Puente, Nadav Friedmann, Lindsay H. Burns

**Affiliations:** ^1^ Department of Molecular, Cellular and Biomedical Sciences, City University of New York School of Medicine, New York, NY, United States; ^2^ Department of Biology and Neuroscience, Graduate School of the City University of New York, New York, NY, United States; ^3^ IMIC, Inc., Palmetto Bay, FL, United States; ^4^ Cognitive Clinical Trials, Omaha, NE, United States; ^5^ Cassava Sciences, Inc., Austin, TX, United States

**Keywords:** filamin A, insulin resistance, mTOR, PTEN, aging, Alzheimer’s disease

## Abstract

**Introduction:** Implicated in both aging and Alzheimer’s disease (AD), mammalian target of rapamycin (mTOR) is overactive in AD brain and lymphocytes. Stimulated by growth factors such as insulin, mTOR monitors cell health and nutrient needs. A small molecule oral drug candidate for AD, simufilam targets an altered conformation of the scaffolding protein filamin A (FLNA) found in AD brain and lymphocytes that induces aberrant FLNA interactions leading to AD neuropathology. Simufilam restores FLNA’s normal shape to disrupt its AD-associated protein interactions.

**Methods:** We measured mTOR and its response to insulin in lymphocytes of AD patients before and after oral simufilam compared to healthy control lymphocytes.

**Results:** mTOR was overactive and its response to insulin reduced in lymphocytes from AD versus healthy control subjects, illustrating another aspect of insulin resistance in AD. After oral simufilam, lymphocytes showed normalized basal mTOR activity and improved insulin-evoked mTOR activation in mTOR complex 1, complex 2, and upstream and downstream signaling components (Akt, p70S6K and phosphorylated Rictor). Suggesting mechanism, we showed that FLNA interacts with the insulin receptor until dissociation by insulin, but this linkage was elevated and its dissociation impaired in AD lymphocytes. Simufilam improved the insulin-mediated dissociation. Additionally, FLNA’s interaction with Phosphatase and Tensin Homolog deleted on Chromosome 10 (PTEN), a negative regulator of mTOR, was reduced in AD lymphocytes and improved by simufilam.

**Discussion:** Reducing mTOR’s basal overactivity and its resistance to insulin represents another mechanism of simufilam to counteract aging and AD pathology. Simufilam is currently in Phase 3 clinical trials for AD dementia.

## Introduction

The neurodegenerative process in Alzheimer’s disease (AD) is characterized by synaptic damage leading to progressively impaired synaptic plasticity and eventual neuronal loss driven by multiple signaling abnormalities, including the overactivation of mammalian target of rapamycin (mTOR) (Guo et al., 2017). Best known as a driver of cancer, mTOR is a critical protein kinase that regulates cell growth and metabolism via two multiprotein complexes (Liu and Sabatini, 2020; Szwed et al., 2021). mTOR complex 1 (mTORC1) is sensitive to rapamycin and contains regulatory-associated protein of mTOR (Raptor); whereas, mTORC2 instead contains rapamyacin-insensitive companion of mTOR (Rictor) (Rosner et al., 2008; Saxton and Sabatini, 2017; Switon et al., 2017; Liu and Sabatini, 2020; Szwed et al., 2021). Controlling energy homeostasis, mTORC1 responds to intracellular and extracellular factors including insulin to regulate protein synthesis, while limiting autophagic breakdown of cellular components (Fingar et al., 2004; Laplante and Sabatini, 2012; Switon et al., 2017; Liu and Sabatini, 2020). mTORC2 can also be activated by growth factors including insulin to regulate cell survival, proliferation, cell mobility and metastasis of cancer cells (Saxton and Sabatini, 2017; Knudsen et al., 2020; Szwed et al., 2021).

With this central role in cellular homeostasis and survival, mTOR, when dysregulated, has been implicated not just in cancer, but also in metabolic syndromes (Yoon, 2017), neurodegeneration, and aging (Rosner et al., 2008; Bloom et al., 2018; Liu and Sabatini, 2020). mTORC1 activation contributes to aging because it accelerates protein synthesis, mitochondrial energy production and resulting oxidative stress, as well as entry into cellular senescence (Liu and Sabatini, 2020; Szwed et al., 2021). By actively suppressing autophagy, mTORC1 contributes to aging by protein accumulation and aggregation (Cai et al., 2012; Kim and Guan, 2015; Perluigi et al., 2015; Liu and Sabatini, 2020). Illustrating mTORC1’s role in aging, its inhibition by rapamycin has been shown to increase life span in mice (Harrison et al., 2009; Miller et al., 2014).

In addition to promoting aging, overactivation of mTORC1 appears to contribute specifically to the neuropathology of AD (Rosner et al., 2008; Cai et al., 2012; Cai et al., 2015). Overactive mTORC1 signaling has been demonstrated in AD postmortem hippocampus by elevated phosphorylated serine^2448^ (pS^2448^) mTOR, indicating mTORC1 activation (Talbot et al., 2012; Tang et al., 2015; Majd and Power, 2018), in mouse models of AD (Caccamo et al., 2010) and in AD patient lymphocytes (Paccalin et al., 2006). Sustained activation of mTOR in AD also disables its activation by insulin, contributing to insulin resistance (Perluigi et al., 2015; Bloom et al., 2018). The overactivation of mTOR signaling in AD is evidenced by dysregulation of PTEN, Akt, S6K and mTOR itself (Griffin et al., 2005; Rosner et al., 2008; Talbot et al., 2012; Cai et al., 2015). Activated mTOR in lymphocytes of AD patients correlates with cognitive decline (Paccalin et al., 2006), suggesting a parallel mTOR activation in brain. This overactive mTORC1 signaling in AD appears related to soluble amyloid β_1-42_ (Aβ_42_), because Aβ_42_ activates the PI3K/Akt pathway, leading to mTORC1 activation (Oddo, 2012). Additionally, overactive mTOR and activated p70S6K (a downstream kinase used to confirm mTORC1 activation) are found in brains of AD transgenic mice (Caccamo et al., 2010) and can be induced by injection of Aβ_42_ into hippocampi of wildtype mice (Caccamo et al., 2011). Finally, rapamycin administered prior to amyloid plaque formation in these mouse models can ameliorate cognitive deficits and slow the accumulation of amyloid plaques and neurofibrillary tangles (Caccamo et al., 2010; Majumder et al., 2011; Caccamo et al., 2013).

The mTORC1 and mTORC2 signaling pathways involve multiple points of activation, crosstalk and regulation (Xie and Proud, 2013). The mTORC1 pathway is often described as the PI3K/Akt/mTOR pathway: PI3K activates Akt, a serine/threonine kinase via phosphorylation at T^308^ by phosphoinositide-dependent kinase1 (PDK1) that activates mTORC1 (Laplante and Sabatini, 2012; Yoon, 2017). However, PI3K can also activate mTORC2 via a positive feedback loop between Akt and mTORC2 (Laplante and Sabatini, 2012; Talbot et al., 2012; Xie and Proud, 2013; Yoon, 2017), and mTORC2 directly activates Akt by phosphorylation at S^473^ (Sarbassov et al., 2005; Xie and Proud, 2013). Hence, mTORC1 activation by Akt is amplified by mTORC2 feedback to further activate Akt and thus mTORC1. mTORC1 activation is indicated by phosphorylation of mTOR at S^2448^ and by phosphorylation of its downstream activation target p70S6K at T^389^, while mTOR phosphorylation at S^2481^ indicates mTORC2 activation (Rosner et al., 2008). Activated p70S6K can phosphorylate Rictor, a key component of mTORC2, at T^1135^. Although it does not affect mTORC2 assembly or kinase activity, a T1135A genetic modification of Rictor increased mTORC2 activation of Akt, suggesting a negative regulation of Akt and consequently of mTORC1 by Rictor phosphorylation (Julien et al., 2010).

Insulin activates mTOR via the insulin receptor’s signaling molecule insulin receptor substrate-1 (IRS-1)’s activation of the PI3K/Akt/mTORC1 pathway. mTORC1’s downstream target p70S6K phosphorylates IRS-1 at multiple inhibitory sites, promoting degradation of this signaling adaptor, thereby contributing to insulin resistance, especially with increasing activation of mTORC1 (Rosner et al., 2008). mTOR overactivation has been observed in obese rats in concert with increased inhibitory phosphorylation of IRS-1, indicating impaired insulin signaling to Akt (Khamzina et al., 2005). Insulin resistance has been demonstrated in AD brain (Talbot et al., 2012), and chronic mTOR activation has been proposed to contribute to insulin resistance (Yoon, 2017).

Currently in Phase 3 clinical trials for AD dementia, small molecule drug candidate simufilam was hypothesized to suppress elevated mTOR activation in AD because it disrupts a predominant AD pathogenic pathway, and because it improves insulin receptor signaling (Wang et al., 2012; Wang et al., 2017). Simufilam binds and restores to normal an altered conformation of the scaffolding protein filamin A (FLNA) (Wang et al., 2017). FLNA is a large intracellular protein of 24 immunoglobulin-like repeats that dimerizes at its C-terminal near the cell membrane and crosslinks actin at its N-terminal (Nakamura et al., 2011). Because FLNA interacts with over 90 different proteins (Nakamura et al., 2011), an altered conformation of FLNA in AD could disrupt its normal protein interactions in addition to inducing aberrant ones. Altered FLNA in AD aberrantly links to the α7 nicotinic acetylcholine receptor (α7nAChR) to enable the toxic signaling of soluble amyloid through this receptor to activate kinases (Dineley et al., 2002; Nagele et al., 2002; Wang et al., 2003) that hyperphosphorylate tau, leading to neurodegeneration, tau-containing tangles, and even intracellular Aβ_42_ aggregates and amyloid plaques (Wang et al., 2012; Wang et al., 2017; Burns et al., 2023). Simufilam disrupts this toxic pathway by dissociating FLNA from α7nAChR and similarly disables soluble amyloid’s activation of a toll-like receptor 4 (TLR4)-mediated neuroinflammation pathway (Wang et al., 2012; Wang et al., 2017; Burns et al., 2023). The improvement in insulin receptor signaling by simufilam (Wang et al., 2012) was hypothesized to affect mTOR and its responsiveness to insulin.

In the current work, we examined the effects of simufilam on mTOR activity in the lymphocytes of AD subjects who had participated in a Phase 2a clinical trial of simufilam. Lymphocytes were utilized because they are accessible and contain FLNA, mTOR and insulin receptors. We previously reported significant improvements in multiple exploratory CSF and plasma biomarkers in this clinical study, as well as simufilam treatment effects on FLNA’s aberrant receptor linkages in lymphocytes (Wang et al., 2020). The reduced linkages of FLNA with α7nAChR and TLR4 in lymphocytes of simufilam-treated patients had been earlier demonstrated in both brains and lymphocytes of AD transgenic mice as well as in postmortem human AD brain (Wang et al., 2017) (lymphocytes of mice unpublished). To assess mTOR activation and response to insulin in patient lymphocytes in the current work, we compared basal and insulin-stimulated levels of five parameters indicating activation of mTORC1 or mTORC2 signaling before and after simufilam treatment. In addition to assessing simufilam treatment effects on mTOR activation and its insulin response, we compared AD lymphocytes prior to treatment to healthy control lymphocytes.

To explore potential mechanisms, we measured FLNA’s linkage to the insulin receptor and its dissociation in response to insulin. We also measured FLNA phosphorylation at S^2152^ and FLNA’s interaction with PTEN. We considered that by restoring FLNA’s normal shape, simufilam might reduce FLNA’s hyperphosphorylation and normalize FLNA’s interaction with PTEN. PTEN is a dual lipid and protein phosphatase and a well-known tumor suppressor that inhibits mTOR. PTEN negatively regulates the phosphoinositide 3 kinase (PI3K)/Akt/mTOR pathway by dephosphorylating PIP3 (Georgescu, 2010). To explore the potential translation of simufilam’s effects on lymphocytes to brain, we used 6 well-matched sets of postmortem AD, amnestic mild cognitive impairment (MCI), non-amnestic MCI and control brain tissue to assess the effect of *ex vivo* simufilam on FLNA’s hyperphosphorylation.

## Materials and methods

### Materials and chemicals

Protease inhibitor cocktail (Complete mini EDTA-free protease inhibitors, Roche, 04693159001), protein phosphatase inhibitor cocktail (Phosphostop phosphatase inhibitors, Roche, 04906837001), anti-mTOR (T-2949) for immunoprecipitation and Histopaque-1077 were purchased from Sigma/Millipore. Anti-mTOR (SC-517464) for Western blotting, -pS^2448^mTOR (SC-293133), -pS^2481^mTOR (SC-293089), -Rictor (SC-271081), -Raptor (SC-81537), -p70S6Kα (SC-8418, SC-393967), -Akt1 (SC-5298, SC-55523), -pS^473^Akt (SC-514032), -FLNA (SC-58764, SC-17749, SC-271440), -IRβ (SC-09), and -PTEN (SC-7941, Cell Signaling #9559) were purchased from Santa Cruz Biotechnology. Anti-pS^2152^FLNA antibody (TA313881) was purchased from Origene Technologies. Anti-pT^1135^Rictor (#3806) and -pT^389^p70S6K (#9205) were purchased from Cell Signaling Technology. Insulin (human recombinant; #12585014), zinc solution (#12585014), covalently conjugated protein A/G-agarose beads (#20423), PageRuler™ Plus Prestained Protein Ladder, 10–250 kDa (#26619) and SuperSignal™ West Pico PLUS Chemiluminescent Substrate (#34577) were purchased from ThermoFisher Scientific.

### Lymphocyte collection and processing

Lymphocytes from AD subjects and age-matched controls, from AD subjects in a clinical trial of simufilam, and from healthy controls of three different age ranges (20–30, 40–50 and 65+) were collected under IRB-approved protocols described below.

To prepare lymphocytes, 8 mL venous blood collected in EDTA-containing tubes was layered onto 8 mL Histopaque-1077 at 25°C and centrifuged (400 g, 30 min, 25°C) to yield plasma (top layer) and lymphocytes (opaque interface). The obtained lymphocytes were washed twice by mixing with 10 mL phosphate-buffered saline (PBS) followed by centrifugation at 250 g for 10 min. The final lymphocyte pellet was resuspended in 600 μl cell freezing medium (DMEM, 5% DMSO, 10% fetal bovine serum), aliquoted and held at −80°C until assay.

### Clinical protocols

All blood samples were collected under IRB-approved protocols and were de-identified to the lab receiving samples. The initial study collecting blood samples from AD and age-matched healthy control subjects (Figures 5A, B, 2 males and 2 females in each group) was approved by Quorum IRB. Both the Phase 2a clinical trial of simufilam and the protocol for collection of healthy control lymphocytes were approved by Advarra. Informed Consent Forms signed by all subjects are retained by the sites. All assays were performed by experimenters blind to AD versus healthy control (Figures 5A, B and 6 data), Day of treatment (Phase 2a trial), and age group (healthy control protocol).

In the first-in-patient clinical trial of simufilam (NCT03748706), 19 subjects screened and 13 enrolled. These were 9 females, 4 males; 3 black, 10 white; 6 Hispanic and 7 non-Hispanic. All 13 were mild-to-moderate AD patients, age 50–85, MMSE ≥ 16 and ≤24, with a CSF total tau/Aβ_42_ ratio ≥ 0.30. Subjects received 100 mg oral simufilam b.i.d. for 28 days. CSF was collected at screening and Day 28. Blood samples for plasma biomarkers and lymphocytes were collected Day 1 before dosing, Day 14 and Day 28.

The collection of whole blood from healthy subjects was conducted later and specifically for comparison to the AD subjects. Lymphocytes were isolated from 18 healthy subjects, 6 each in three different age groups (20–30, 40–50 and 65+). These included 12 females and 6 males; 13 white, 2 Asian, 2 American Indian and 1 black participant. Age-matched controls (65+) and two younger cohorts were included so that age differences among healthy controls could be assessed. There were no significant or notable differences between age groups of healthy control subjects on any of the five mTOR parameters due to within group variability and the small group size (*n* = 6). Because there were no age differences, these age ranges were combined into one healthy control group (*n* = 18).

### Lymphocyte incubation and prep for immunoprecipitation and immunoblotting

Lymphocytes (200 μg) from AD patients or healthy controls were incubated at 37°C with oxygenated protease inhibitors containing Kreb’s-Ringer (K-R) or either 0.1 μM Aβ_42_ for 30 min (for experiment to assess FLNA phosphorylation at S^2152^ or PTEN—FLNA linkage) or 1 nM insulin for 15 min (for experiments to assess FLNA linkage to IRβ or mTOR activation). Total incubation volumes were 250 μl. Assay mixtures were aerated with 95%O_2_/5%CO_2_ for 1 min every 10 min. Reactions were terminated by adding ice-cold Ca^2+^-free K-R containing protease and protein phosphatase inhibitors and centrifuged. Lymphocytes were then homogenized in 250 μl ice-cold immunoprecipitation buffer containing 25 mM HEPES, pH 7.5, 200 mM NaCl, 1 mM EDTA, 0.2% 2-mercaptoethanol, and protease and protein phosphatase inhibitors by sonication for 10 s on ice and solubilized by nonionic detergents: 0.5% NP-40/0.2% Na cholate/0.5% digitonin for 60 min (4°C) with end-to-end rotation. The obtained lysates were cleared by centrifugation at 20,000 g for 30 min (4°C) and the resultant supernatants (0.25 ml) were diluted 4x with 0.75 ml immunoprecipitation buffer for immunoprecipitation and immunoblotting as described below.

To detect phosphorylation of a protein, the protein of interest was immunoprecipitated by a phospho-independent antibody, and the specific phospho-epitope was detected by immunoblotting with a phospho-specific antibody. In the case of co-immunoprecipitation, the first protein was immunoprecipitated, and the interacting protein was detected in the immunoprecipitate by immunoblotting. For all immunoblotting experiments, bands were quantified by densitometric quantitation, and these values for all bands in each group were analyzed statistically. Not all groups were run on the same blots; however, each lane was normalized to its own loading control, i.e., the protein that was immunoprecipitated (mTOR, p70S6K, Akt1 or FLNA). With these internal controls, separate blots and experiments conducted separately can be compared.

### Assessment of simufilam treatment on FLNA phosphorylation at pS^2152^ and levels of associated PTEN

*Ex vivo* Aβ_42_ treatment and determination of levels of pS^2152^FLNA and FLNA-associated PTEN were assessed in lymphocytes from healthy controls and age-matched AD patients. After incubating lymphocytes with K-R or 0.1 μM Aβ_42_ for 30 min and homogenizing and solubilizing as described above, FLNA-associated protein complexes were immunoprecipitated with immobilized anti-FLNA (SC-58764 + SC-271440) antibodies onto covalently protein A/G-conjugated agarose beads. The resultant immunocomplexes were pelleted by centrifugation (4°C), washed three times with ice-cold PBS, pH 7.2, containing 0.1% NP-40, and centrifuged again. Immunocomplexes were then solubilized by boiling for 5 min in 100 μl SDS-PAGE sample preparation buffer (62.5 mM Tris-HCl, pH 6.8; 10% glycerol, 2% SDS; 5% 2mercaptoethanol, 0.1% bromophenol blue) and centrifuged to remove antibody-protein A/G agarose beads. Levels of pS^2152^FLNA and PTEN were determined by immunoblotting first with anti-pS^2152^FLNA. Blots were then stripped and re-probed with specific antibodies against PTEN (SC-7974) and FLNA (SC-17749) to access associated PTEN levels and ascertain immunoprecipitation efficiency and gel loading, respectively (Wang et al., 2017).

In a separate experiment, anti-FLNA immunoprecipitates of lymphocyte lysates of the phase 2a subjects were used to assess the effects of simufilam on pS^2152^FLNA and PTEN levels by specific antibodies against pS^2152^FLNA and PTEN (Cell Signaling #9559). Parallel sets of blots with identical amounts of anti-FLNA immunoprecipitates were used to confirm equal loading.

### Assessment of FLNA linkages to IRβ

Lymphocytes from AD patients and healthy controls were assessed for basal and insulin-stimulated FLNA linkage to IRβ. After incubating lymphocytes with K-R or 1 nM insulin for 15 min and homogenizing and solubilizing as described above, FLNA-associated protein complexes were immunoprecipitated with immobilized anti-FLNA (SC-58764 + SC-271440) antibodies onto covalently protein A/G-conjugated agarose beads. The resultant immunocomplexes were pelleted by centrifugation (4°C), washed three times with ice-cold PBS, pH 7.2, containing 0.1% NP-40, and centrifuged again. Immunocomplexes were then solubilized by boiling for 5 min in 100 μl SDS-PAGE sample preparation buffer (described above) and centrifuged to remove antibody-protein A/G agarose beads. Levels of IRβ were determined by immunoblotting with anti-IRβ. Blots were stripped and re-probed with specific antibodies against FLNA (SC-17749) to indicate immunoprecipitation efficiency and gel loading (Wang et al., 2017). For healthy control lymphocytes, levels of IRβ and FLNA were determined by immunoblotting with anti-IRβ and anti-FLNA (SC-17749) simultaneously.

### Assessments of basal and insulin-stimulated mTOR activation

Lymphocytes from AD patients and healthy controls were assessed for basal and insulin-stimulated mTOR activation. After incubating lymphocytes with K-R or 1 nM insulin for 15 min and homogenizing and solubilizing as described above, protein complexes were immunoprecipitated with immobilized anti-mTOR (T-2949), -Akt1 (SC-5298, SC-55523), or -p70S6K (SC-8418, SC-393967) antibodies onto covalently protein A/G-conjugated agarose beads. Immunocomplexes were pelleted by centrifugation (4°C), washed three times with ice-cold PBS, pH 7.2, containing 0.1% NP-40, and centrifuged again. Immunocomplexes were then solubilized by boiling for 5 min in 100 μl SDS-PAGE sample preparation buffer (described above) and centrifuged to remove antibody-protein A/G agarose beads. Activated mTORC1 was defined by the levels of pS^2448^mTOR in the anti-mTOR immunoprecipitates by immunoblotting and supported by pT^389^p70S6K in anti-p70S6K immunoprecipitates and pT^1135^Rictor in mTOR immunoprecipitates. mTORC1—Raptor linkage was assessed by immunoblotting with anti-Raptor. The activity of mTORC2 was defined by the levels of pS^2481^mTOR and supported by pS^473^Akt1 in the anti-Akt1 immunoprecipitates. mTORC2—Rictor association was determined by the levels of Rictor in the anti-mTOR immunoprecipitate by immunoblotting with anti-Rictor. Blots were stripped and re-probed with respective specific antibodies against mTOR (SC-517464), p70S6K (SC-393967) or Akt1 (SC-5298) to indicate immunoprecipitation efficiency and gel loading.

### Assessment of pS^2152^FLNA in postmortem human AD brain tissue

Using an established method (Wang et al., 2012), pS^2152^FLNA and IRβ proteins in synaptosomes from Aβ_42_-incubated hippocampal slices from 6 sets of age (81–94 years) and postmortem interval (2–8.8 h) matched control, amnestic MCI, non-amnestic MCI and AD subjects (4 females/2 males) with and without 1 nM simufilam were immunoprecipitated with immobilized anti-FLNA (SC-7565). Briefly, hippocampal slices were incubated with K-R, 1 nM simufilam or 1 μM Aβ_42_ at 37°C for 30 min. Synaptosomes (200 μg) from K-R, simufilam or Aβ_42_ incubated postmortem hippocampal slices were pelleted by centrifugation, solubilized by brief sonication in 250 μL of immunoprecipitation buffer (25 mM HEPES, pH 7.5; 200 mM NaCl, 1 mM EDTA, cocktail of protease, and protein phosphatase inhibitors) and incubated at 4°C with end-to-end shaking for 1 h. Following dilution with 750 μL of ice-cold immunoprecipitation buffer and centrifugation (4°C) to remove insoluble debris, the FLNA-IRβ complexes in the lysate were isolated by immunoprecipitation with 16-h incubation at 4°C with respective rabbit anti-FLNA (1 μg) immobilized on protein A/G-conjugated agarose beads. The resultant immunocomplexes were pelleted by centrifugation at 4°C. After 3 washes with 1 mL of ice-cold PBS (pH 7.2) and centrifugation, the isolated FLNA-IR complexes were solubilized by boiling for 5 min in 100 mL of SDS-polyacrylamide gel electrophoresis (PAGE) sample preparation buffer (62.5 mM Tris-HCl, pH 6.8; 10% glycerol, 2% SDS; 5% 2-mercaptoethanol, 0.1% bromophenol blue). Levels of pS^2152^FLNA and the content of IRβ in 50% of the anti-FLNA immunoprecipitates were determined by immunoblotting with purified rabbit anti-pS^2152^FLNA (TA313881, Origene) and mouse monoclonal anti-IRβ (SC-81465) antibodies. Blots were stripped and re-probed with monoclonal anti-FLNA (SC-271440) to validate equal immunoprecipitation efficiency and loading.

### Statistics

All data were analyzed by an independent statistician using SAS, except for Figures 5, 6, which were analyzed in Excel. Comparisons between healthy controls and AD at Day 0 used the two-sided two-sample *t*-test (and were not notably different from analyses using the non-parametric Wilcoxon-Mann-Whitney test). Within-subject comparisons between Day 0 and Day 14 or Day 0 and Day 28 were analyzed by two-sided paired *t*-test (and were not notably different from results using non-parametric tests Wilcoxon signed rank or sign test). Because parametric and non-parametric analyses did not produce notably different results, distributions were assumed to be normal, justifying the use of the parametric *t*-test.

## Results

Multiple measures of mTORC1 and mTOR2 activation showed elevated basal activity in AD lymphocytes compared to healthy control lymphocytes. The elevated basal activity was paired with very little stimulation by *in vitro* insulin across these measures. Simufilam administered orally to the AD patients reduced the heightened basal activity and enhanced sensitivity to insulin. The first three subsections below show indicators of mTORC1 activation, mTORC2 activation, and Akt1 activation, the latter in the mTORC1 pathway but also stimulated by mTORC2. The fourth subsection reports FLNA’s interaction with the insulin receptor, which is dissociated in response to insulin, perhaps to allow recruitment of IRS-1. The FLNA—insulin receptor interaction is higher in AD lymphocytes, and its dissociation by insulin is impaired, but improved by simufilam. The fifth subsection shows another possible mechanistic explanation for the elevated mTOR activation: FLNA linkage to the mTOR suppressor PTEN was reduced in AD lymphocytes compared to healthy controls, coincident with elevated phosphorylation of FLNA at S^2152^. Simufilam oral treatment restored the FLNA—PTEN linkage and reduced FLNA’s hyperphosphorylation. In the final subsection, elevated pS^2152^FLNA is also shown in postmortem AD and amnestic MCI brain, and this hyperphosphorylation was reduced by *ex vivo* incubation with simufilam.

### Simufilam reduced basal mTORC1 signaling and restored its insulin sensitivity

**FIGURE 1:**
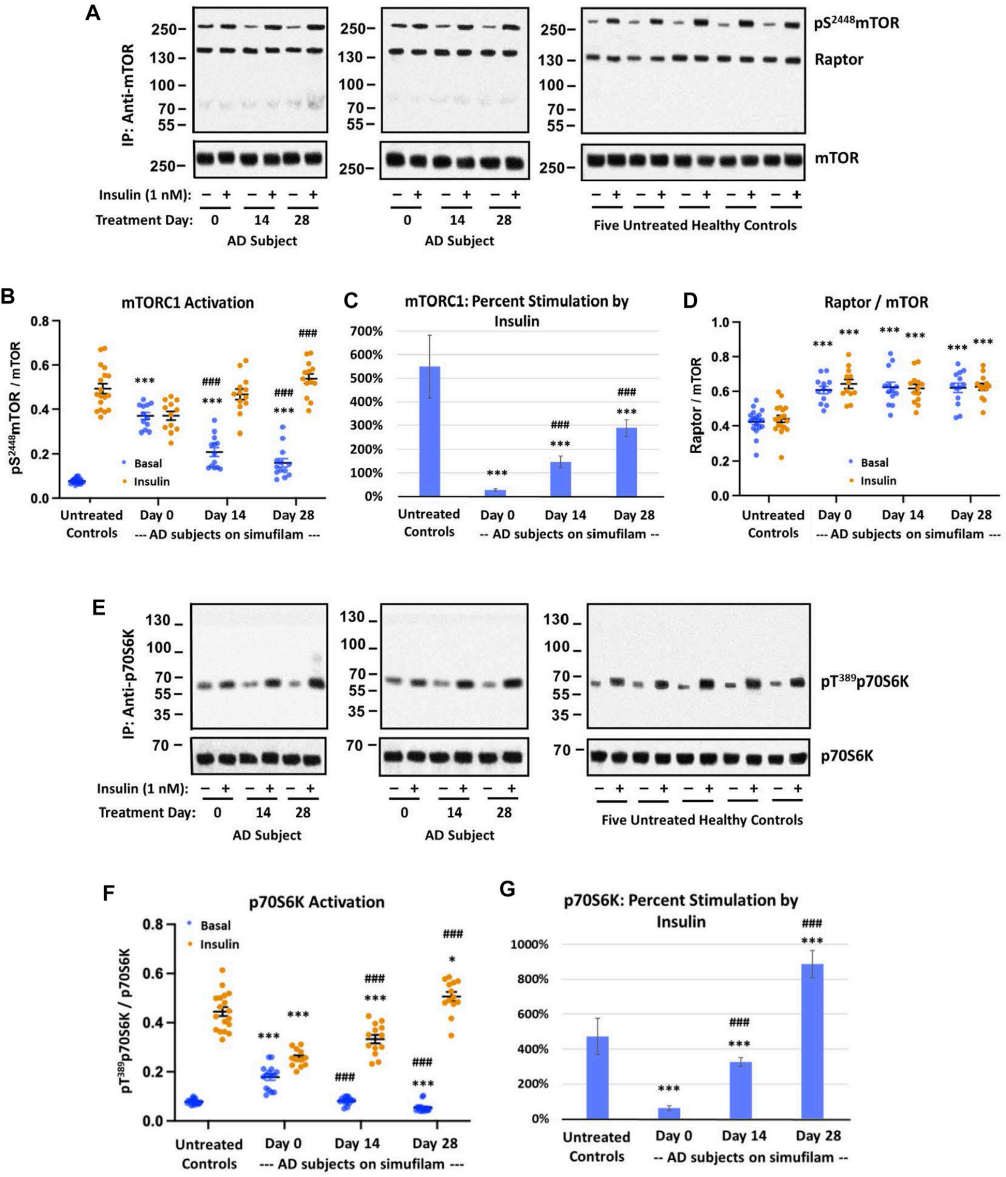
Simufilam treatment attenuated basal mTORC1 overactivation and restored its response to insulin in lymphocytes from AD subjects. Representative blot images from two AD and five healthy control subjects **(A,E)** show increased basal activation of mTORC1 by elevated levels of mTOR phosphorylation at S^2448^ **(A)** and by increased basal activation of mTORC1’s downstream kinase p70S6K, indicated by phosphorylation at T^1389^ **(E)**, in AD subjects versus healthy controls. Blots were analyzed by densitometric quantitation **(B,D,F)**. With heightened basal activity of the mTORC1 signaling pathway in AD subject lymphocytes, its response to *in vitro* insulin was blunted. The elevated basal p70S6K, still lower than insulin-stimulated control levels, also showed a weak response to insulin. Oral simufilam treatment decreased the elevated basal mTORC1 activation and restored its response to insulin across both measures, with insulin-stimulated p70S6K levels slightly higher than untreated healthy controls on Day 28. The simufilam treatment effect is also seen in the dramatic improvement in percent stimulation by insulin **(C,G)**. mTOR association with Raptor was lower in healthy controls but unaffected by insulin or simufilam **(D)**. Data are means ± SEM. *N* = 13 for AD, 18 for control. *p < 0.05, ***p < 0.001, vs. respective value in healthy control by two-sided two-sample *t*-test; ^###^ *p* < 0.001 vs. respective AD value at Day 0 by two-sided paired *t*-test.

Basal mTORC1 signaling was elevated and its response to insulin was blunted in lymphocytes from AD compared to healthy control subjects. These differences were attenuated after simufilam treatment (Figure 1). Basal activity of mTORC1, indicated by levels of pS^2448^mTOR, was significantly elevated in AD subjects’ lymphocytes relative to healthy controls (Figures 1A, B; *p* < 0.001). This heightened basal activity in AD lymphocytes was nearly as high as the insulin-stimulated level in healthy controls and was not further stimulated by insulin. Simufilam treatment of AD subjects significantly reduced this heightened basal mTORC1 activity by Day 14 and Day 28 (*p* < 0.001). The level of mTORC1 activation following insulin stimulation was not different from healthy controls at any treatment day for AD subjects, although the Day 28 insulin-stimulated mTORC1 activation level was significantly higher than at Day 0 for AD subjects (*p* < 0.001). Notably, percent stimulation by insulin of mTORC1 was markedly reduced in AD subjects before simufilam treatment relative to healthy controls (Figure 1C; *p* < 0.001). Insulin responsiveness significantly improved on Day 14 and Day 28 of treatment (*p* < 0.001).

These changes in mTORC1 activation in AD subjects and improvements following simufilam treatment were not due to changes in mTOR or Raptor expression levels or association of mTOR with Raptor, a component of mTORC1. Interestingly however, mTOR—Raptor association was lower in healthy control than in AD subject lymphocytes (Figure 1D).

Additional confirmation of mTORC1 activation is provided by activation of p70S6K, a downstream kinase of mTORC1 by its phosphorylation at T^1389^. Similar to mTORC1 activation, p70SK6 basal activity, indicated by pT^1389^p70S6K, was elevated in AD subjects relative to healthy controls (Figures 1E, F; *p* < 0.001). This elevated basal p70S6K activity was reduced on Day 14 and Day 28 compared to pre-treatment levels (*p* < 0.001). Insulin-stimulated activation of p70S6K was significantly lower than in healthy controls (*p* < 0.001) before treatment and at Day 14 but was slightly higher than in healthy controls after 28 days of simufilam (*p* < 0.05). Insulin responsiveness of p70S6K was significantly improved at Day 14 and Day 28 (*p* < 0.001). Percent stimulation by insulin was significantly lower in AD subjects before treatment and on Day 14 compared to healthy controls but significantly higher at Day 28 (Figure 1G; *p* < 0.001).

### Simufilam reduced basal mTORC2 signaling and restored its insulin sensitivity

**FIGURE 2:**
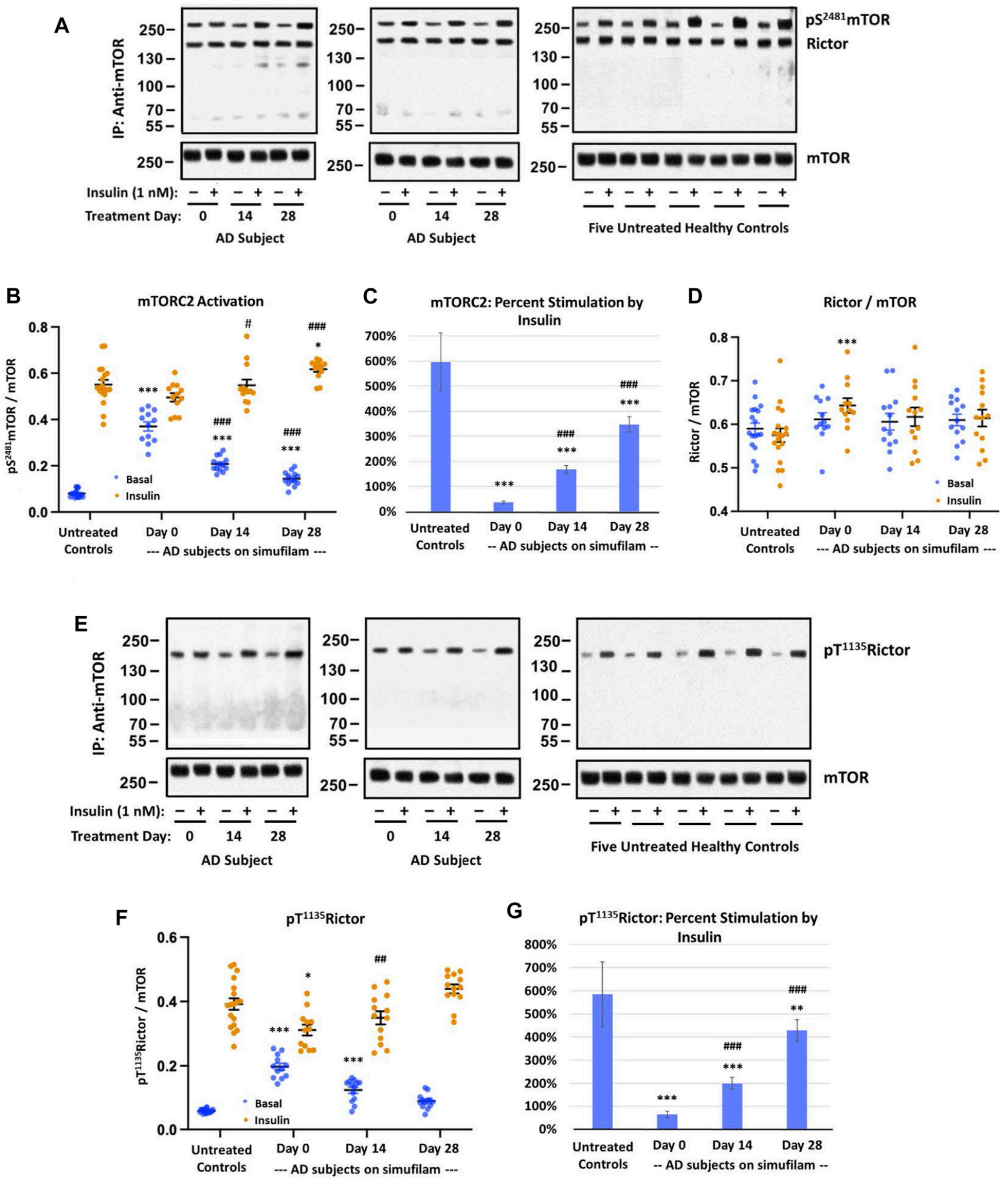
Simufilam treatment attenuated basal mTORC2 overactivation and restored its response to insulin in lymphocytes from AD subjects. Representative blot images from two AD and five healthy control subjects **(A,E)** show increased basal mTORC2 signaling by elevated levels of mTOR phosphorylation at S^2481^ **(A)** and by increased phosphorylation of the mTORC2 component Rictor at T^1135^ **(E)** in AD subjects versus healthy controls. Blots were analyzed by densitometric quantitation **(B,D,F)**. As with mTORC1 signaling, the heightened basal mTORC2 signaling in AD subjects left little room for insulin stimulation. The elevated levels of pT^1135^Rictor, still lower than insulin-stimulated control levels, again showed a weak response to insulin. Oral simufilam treatment decreased the elevated basal mTORC2 signaling and restored its response to insulin on both measures. Insulin-stimulated p S^2481^mTOR was slightly higher on Day 28 than in untreated healthy controls. Percent stimulation of these mTORC2 signaling components by insulin improved dramatically after simufilam treatment **(C,G)**. mTOR association with Rictor was unchanged in AD and unaffected by insulin or simufilam **(D)**. Data are means ± SEM. *N* = 13 for AD, 18 for control. *p < 0.05, **p < 0.01, ***p < 0.001 vs. respective value in healthy control by two-sided two-sample *t*-test; ^##^ *p* < 0.01, ^###^ *p* < 0.001 vs. respective AD value at Day 0 by two-sided paired *t*-test.

Similar to mTORC1, mTORC2 basal activation was elevated in AD subjects relative to healthy controls, as assessed by levels of pS^2481^mTOR (Figures 2A, B; *p* < 0.001), and this high basal activity was significantly reduced by simufilam treatment (*p* < 0.001). Insulin-induced mTORC2 activation was elevated on Day 14 (*p* < 0.05) and Day 28 (*p* < 0.001) in AD subjects relative to Day 0. Similar to p70S6K, insulin-stimulated mTORC2 on Day 28 of simufilam treatment in AD subjects was slightly higher than in healthy controls (*p* < 0.05). Like mTORC1, percent stimulation by insulin for mTORC2 was markedly reduced in AD subjects at all treatment days relative to healthy controls but markedly increased following simufilam treatment (Figure 2C; *p* < 0.001).

There were no notable differences in mTOR and Rictor expression levels and mTOR—Rictor association between AD and healthy controls and no effects of insulin or simufilam on this interaction, despite a slight increase in insulin-stimulated mTORC2 levels in AD on Day 0 (Figure 2D).

Activation of the mTORC2 component Rictor is evidenced by its phosphorylation at T^1135^. Basal levels of pT^1135^Rictor were elevated in AD compared to healthy control lymphocytes (Figures 2E, F; *p* < 0.001). This heightened level of pT^1135^Rictor was reduced after 14 and 28 days of simufilam treatment (*p* < 0.001). Insulin-stimulated pT^1135^Rictor was slightly lower in untreated AD subjects than in healthy controls (*p* = 0.05) and was significantly increased following simufilam treatment (*p* < 0.01 at Day 14 and *p* < 0.001 at Day 28 vs. Day 0). The slight increase in insulin-stimulated pT^1135^Rictor in AD subjects on Day 28 versus healthy controls is not significant (*p* = 0.07). As with other mTORC1 and mTORC2 markers, percent stimulation by insulin of pT^1135^Rictor was dramatically reduced in AD versus healthy control lymphocytes (Figure 2G; *p* < 0.001) and significantly improved with simufilam treatment (*p* < 0.001).

### Simufilam reduced basal Akt1 activation and restored its insulin sensitivity

**FIGURE 3:**
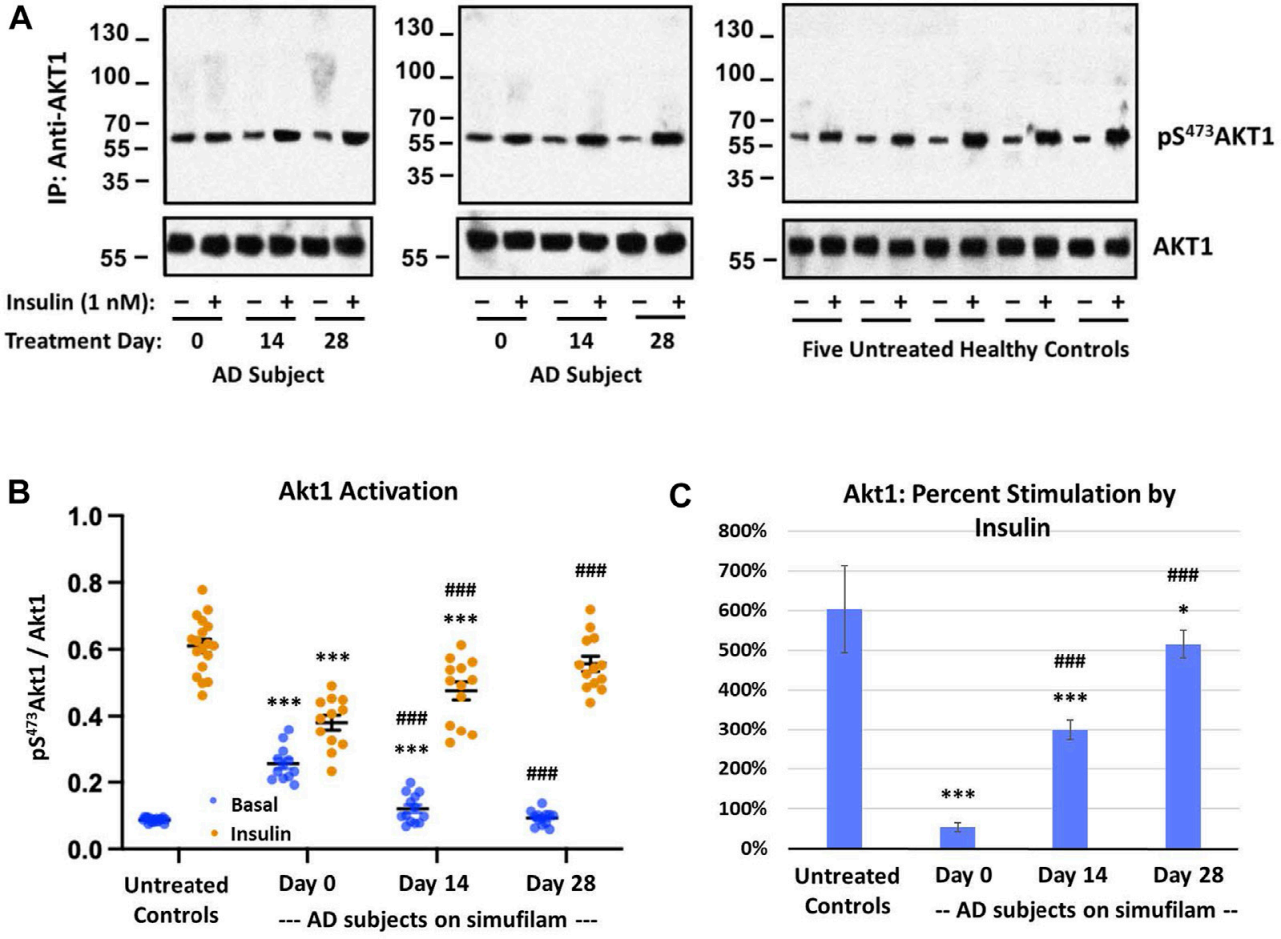
Simufilam treatment attenuates mTORC2-mediated Akt1 activation. Akt1 is activated by mTORC2 signaling by phosphorylation on S^473^ to further activate mTORC1. Representative blot images from two AD and five healthy control subjects **(A)** show increased basal Akt1 activation, as indicated by its phosphorylation at S^473^, in AD subjects versus healthy controls. Blots were analyzed by densitometric quantitation **(B)**. Showing a similar pattern to downstream effectors p70S6K and pT^1135^Rictor, basal Akt1 activity was elevated in AD subjects without nearing insulin-stimulated control levels, and its response to insulin was still blunted. Oral simufilam treatment decreased the elevated Akt1 activation and restored its response to insulin to a level not different from healthy control. Percent stimulation of Akt1 by insulin improved dramatically after simufilam treatment **(C)**. Data are means ± SEM. *N* = 13 for AD, 18 for control. *p < 0.05, ***p < 0.001 vs. respective value in healthy control by two-sided two-sample *t*-test; ^#^ *p* < 0.05, ^###^ *p* < 0.001 vs. respective AD value at Day 0 by two-sided paired *t*-test.

Akt is activated by PI3K in the PI3K/Akt/mTORC1 pathway (indicated by pT^308^Akt) but also by mTORC2 in a positive feedback loop (indicated by pS^473^Akt). Activation of Akt is indicated by its phosphorylation at S^473^. Like the other mTORC1 and mTORC2 markers, basal pS^473^Akt1 was elevated in AD versus healthy control subjects (Figure 3, *p* < 0.001). The heightened basal Akt1 activity was reduced by simufilam treatment (*p* < 0.001) and not different from healthy controls by Day 28. Insulin-stimulated pS^473^Akt1 was also lower in AD than in healthy controls (*p* < 0.001) and improved with simufilam treatment (*p* < 0.001) to a level not different from control on Day 28. Percent stimulation by insulin was once again markedly reduced in AD compared to controls (Figure 3C; *p* < 0.001) and significantly improved with simufilam treatment (*p* < 0.001) to a level close to control on Day 28.

### Simufilam improved dissociation of FLNA—IRβ linkage by insulin

**FIGURE 4:**
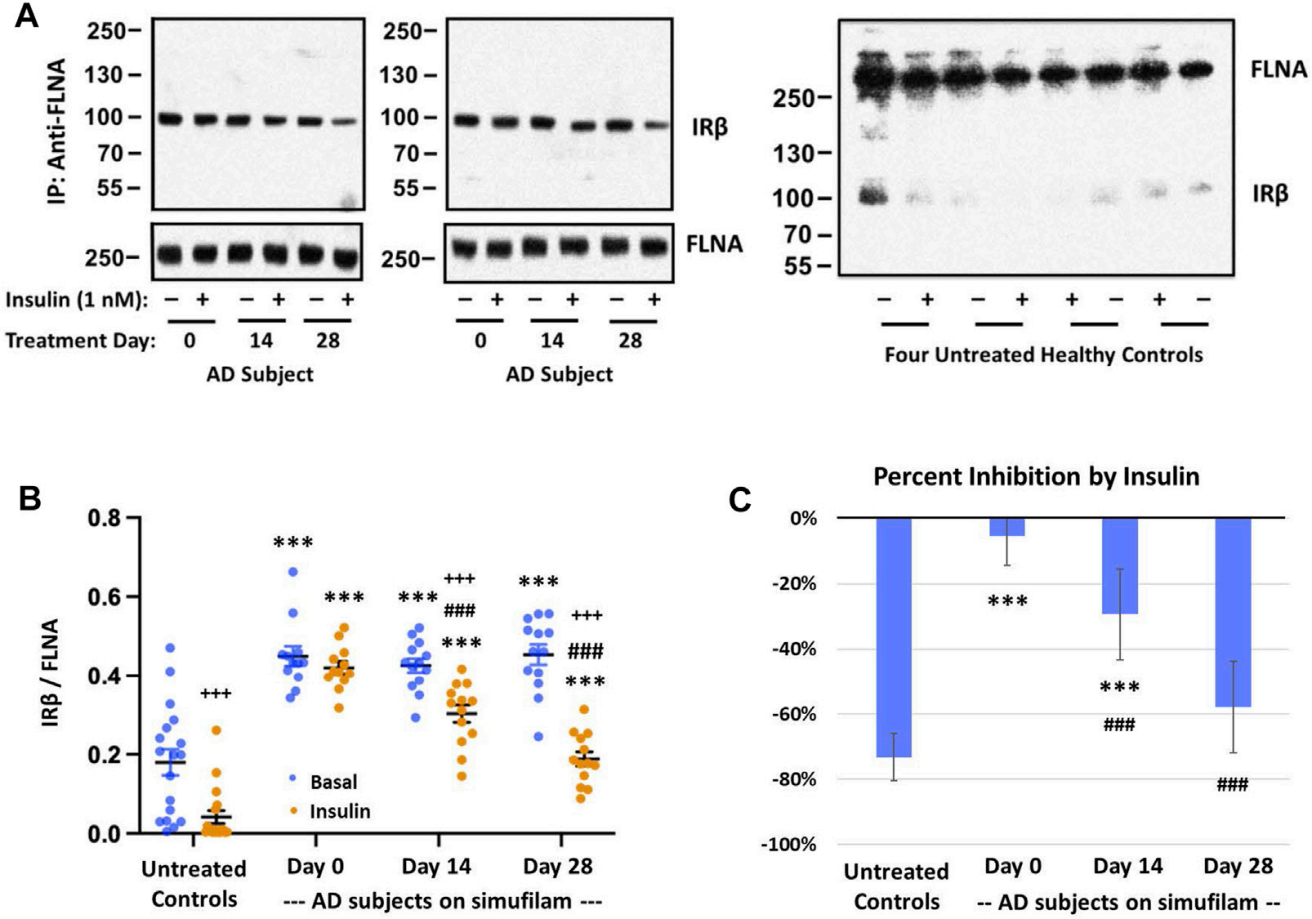
Simufilam treatment restores the insulin-stimulated reduction in FLNA linkage to insulin receptor beta (IRβ). Representative blot images from two AD and four healthy control subjects **(A)** show higher levels of the FLNA—IRβ interaction and impaired dissociation of this linkage upon insulin stimulation in AD subjects versus healthy controls. Simufilam treatment improved the insulin-stimulated dissociation of FLNA—IRβ interaction. Blots were analyzed by densitometric quantitation **(B)**. Percent inhibition by insulin in AD subjects improved dramatically with simufilam treatment **(C)**. Data are means ± SEM. *N* = 13 for AD, 18 for control. ***p < 0.001 vs. respective value in healthy control by two-sided two-sample *t*-test; ^###^ *p* < 0.001 vs. respective AD value at Day 0 by two-sided paired *t*-test; +++*p* < 0.001 insulin vs. basal value at respective timepoint by two-sided paired *t*-test.

To investigate a potential mechanism of simufilam on insulin responsiveness, we examined interactions of simufilam’s target protein, FLNA, with the insulin receptor. Levels of FLNA association with the IRβ subunit of the insulin receptor were lower in healthy control lymphocytes than in AD lymphocytes (Figures 4A, B; *p* < 0.001), as seen by the very faint IRβ bands in the immunoblots of the FLNA immunoprecipitates of healthy control lymphocytes. This linkage was further reduced in response to insulin in the healthy controls (*p* < 0.001), although the magnitude of reduction as calculated by densitometric quantitation of the already faint immunoblot bands may not be reliable. The much higher levels of FLNA linked to IRβ in AD patient lymphocytes suggest it is a pathogenic interaction, perhaps preventing IRβ from interacting with IRS-1. Insulin had very little effect on these much higher levels of FLNA—IRβ association in lymphocytes of AD subjects prior to simufilam treatment (*p* = 0.06). After simufilam treatment, insulin’s ability to reduce this FLNA—IRβ linkage was partially restored on Day 14 and further improved by Day 28 (*p* < 0.001 for Day 14 or 28 vs. Day 0). Percent stimulation by insulin was significantly lower in untreated AD subjects than in healthy controls, and significantly improved with simufilam (Figure 4C; *p* < 0.001) to levels close to healthy control.

### Simufilam reduced pS^2152^FLNA and restored FLNA—PTEN linkage

**FIGURE 5:**
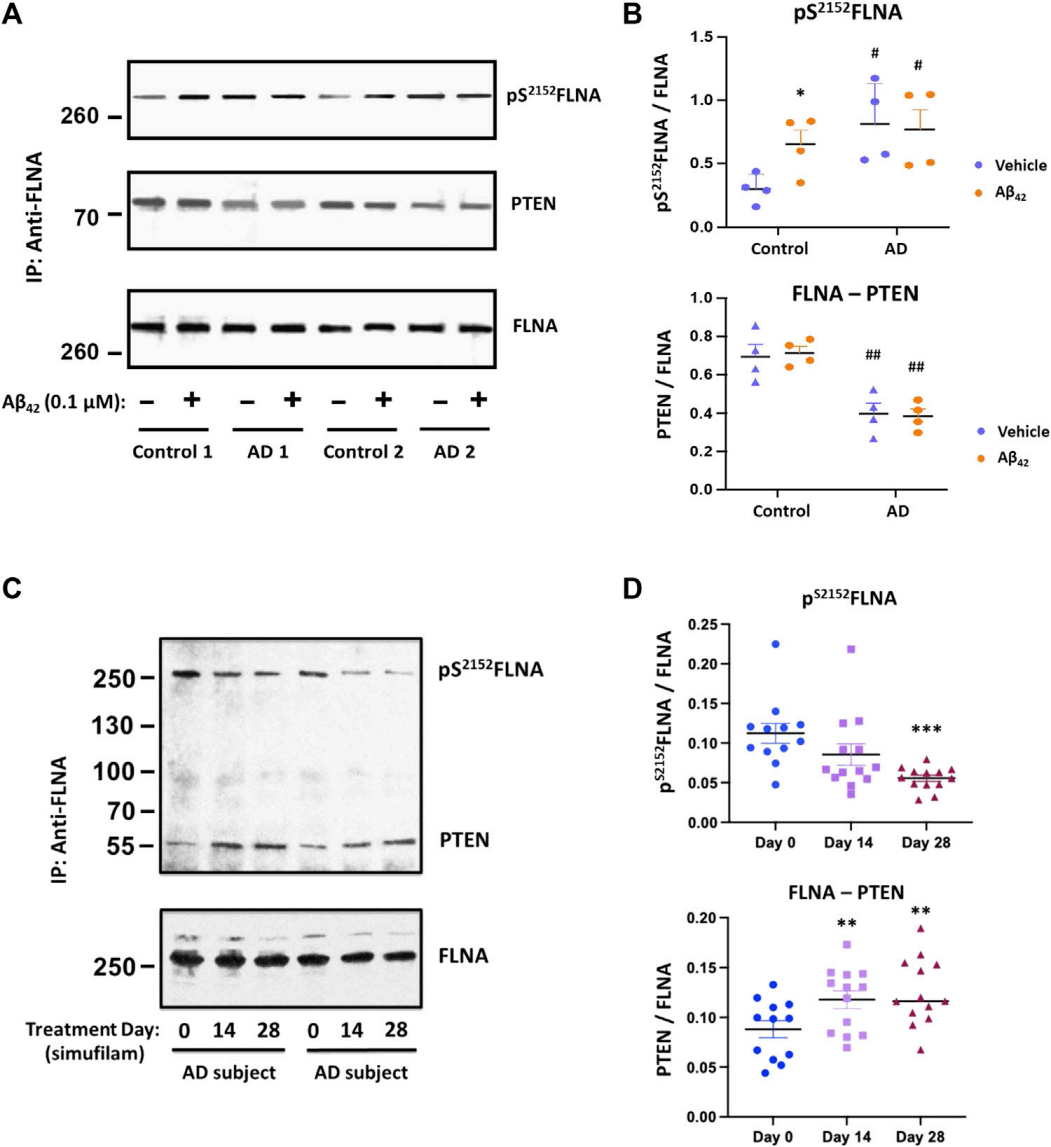
Elevated pS^2152^FLNA and reduced FLNA—PTEN linkage in lymphocytes of AD subjects were improved by oral simufilam. Increased levels of FLNA phosphorylation at S^2152^ were coincident with a reduced FLNA—PTEN linkage in AD versus age-matched control lymphocytes. Exogenous Aβ_42_ increased S^2152^FLNA to AD-like levels but did not reduce the FLNA—PTEN linkage in control lymphocytes. Aβ_42_ had no further effect on AD lymphocytes. [**(A)** representative blot images; **(B)** densitometric quantitation of blot images]. Oral simufilam to AD subjects reduced the high levels of S^2152^FLNA and improved PTEN tethering to FLNA in their lymphocytes [**(C)** representative blot images; **(D)** densitometric quantitation of blot images]. Data are means ± SEM. *N* = 4 for AD and age-matched healthy control **(A,B)**, and *N* = 13 for AD patients treated with simufilam **(C,D)**. *p < 0.05 vs. vehicle by two-sided paired *t*-test **(B)**; ^#^ *p* < 0.05, ^##^ *p* < 0.01 vs. control by two-sided two-sample *t*-test **(B)**; ***p < 0.001, **p < 0.01 vs. Day 0 by two-sided paired *t*-test **(D)**.

To explore additional potential FLNA-mediated mechanisms of the mTOR overactivation and resistance to insulin, we examined FLNA phosphorylation and a critical protein interaction. Specifically, FLNA was hyperphosphorylated at S^2152^ in lymphocytes of AD subjects compared to lymphocytes from age-matched controls (Figures 5A, B; *p* < 0.01). Importantly, incubation of control lymphocytes with 0.1 μM Aβ_42_ for 1 h increased pS^2152^FLNA (*p* < 0.01) to a level comparable to pS^2152^FLNA in AD lymphocytes, suggesting that this FLNA alteration is mediated by Aβ_42_. Correlating with the FLNA hyperphosphorylation, FLNA’s interaction with the mTOR suppressor PTEN was reduced in AD versus control lymphocytes (*p* < 0.01). However, unlike the induction of pS^2152^FLNA by exogenous Aβ_42_, incubation of control lymphocytes with Aβ_42_ did not reduce the FLNA—PTEN interaction to levels comparable to AD lymphocytes, suggesting that the time needed to dissociate PTEN tethering to FLNA may be longer than the time needed to hyperphosphorylate FLNA in response to Aβ_42_. Alternatively, the reduced FLNA—PTEN interaction in AD lymphocytes might have other influences. AD lymphocytes were also incubated with 0.1 μM Aβ_42_ to maintain experimenter blinding and to show that FLNA hyperphosphorylation in AD lymphocytes is mediated by endogenous Aβ_42._ Oral simufilam given to AD subjects in the clinical trial reduced levels of pS^2152^FLNA at Day 28 (Figures 5C, D; *p* < 0.001) and increased FLNA—PTEN linkage in lymphocytes at both Day 14 and Day 28 (*p* < 0.01).

### Simufilam incubation reduced pS^2152^FLNA in AD and amnestic MCI postmortem brain tissue

**FIGURE 6:**
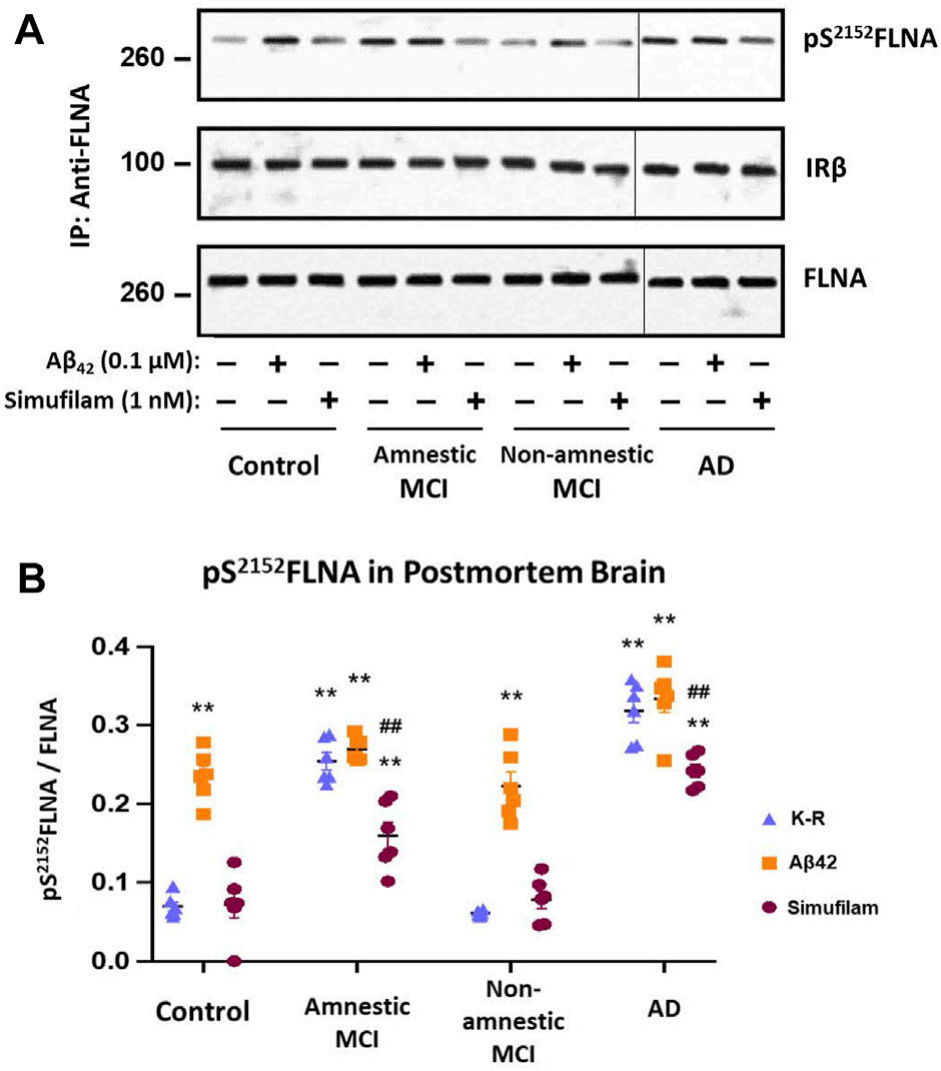
Simufilam reduced S^2152^ phosphorylation of FLNA in postmortem human AD and amnestic MCI brain tissue. FLNA is phosphorylated at S^2152^ in postmortem brain tissue of AD and amnestic MCI subjects compared to non-amnestic MCI and age-matched healthy control subjects [**(A)** representative immunoblot images; **(B)** densitometric quantitation of blot images]. *Ex vivo* incubation with simufilam (1 nM) reduced levels of pS^2152^FLNA in AD and amnestic MCI with no effect on the already low levels in non-amnestic MCI and age-matched healthy control brain tissue. In contrast, incubation with Aβ_42_ increased pS^2152^FLNA levels in non-amnestic MCI and age-matched healthy control subjects but did not further increase the elevated levels in AD or amnestic MCI. Vertical lines indicate separation between 2 blots (9 lanes of one blot with Controls, Amnestic MCI and Non-amnestic MCI and 3 lanes of another blot with AD). Data are means ± SEM. *N* = 6. **p < 0.01 vs. Kreb’s-Ringer (K-R) in control group by two-sided two-sample *t*-test; ^##^ *p* < 0.01 vs. K-R in respective group by two-sided paired *t*-test.

To explore whether simufilam’s effects on lymphocytes may also occur in brain, we examined the FLNA hyperphosphorylation in six well-matched sets of postmortem brain tissue from healthy control, amnestic MCI, non-amnestic MCI and AD subjects. FLNA is hyperphosphorylated at S^2152^ in AD and amnestic MCI compared to control and non-amnestic MCI, as seen by increased levels of pS^2152^FLNA in FLNA immunoprecipitates of solubilized synaptosomes from the brain tissue (Figure 6, *p* < 0.01). Incubation with 1 nM simufilam for 1 h reduced pS^2152^FLNA in AD and amnestic MCI postmortem brain tissue (*p* < 0.01) but had no effect on the already low levels in non-amnestic MCI or control tissue. In reciprocal fashion, incubation of control and non-amnestic MCI tissue with 0.1 μM Aβ_42_ increased pS^2152^FLNA to levels comparable to AD and amnestic MCI (*p* < 0.01) but had no further effect on the already high levels in amnestic MCI and AD tissue. The increase in pS^2152^FLNA by exogenous Aβ_42_ to levels resembling those in AD or amnestic MCI again suggests that this AD-related hyperphosphorylation of FLNA is Aβ_42_-dependent. In contrast to our observations in AD and control lymphocytes, levels of IRβ linked to FLNA were not different between groups and were also not affected by simufilam or Aβ_42_ incubation.

## Discussion

Simufilam is an AD drug candidate that disrupts an upstream pathogenic pathway that hyperphosphorylates tau, prior to the aggregation and deposition of tau or amyloid, as well as a separate neuroinflammatory pathway (Wang et al., 2012; Burns and Wang, 2017; Wang et al., 2017; Wang et al., 2020; Burns et al., 2023). We now show that oral simufilam reduces overactive mTOR signaling in AD lymphocytes, with stronger effects with longer treatment. We found that the interaction of simufilam’s target, FLNA, with the IRβ subunit of insulin receptors is elevated in AD lymphocytes. This interaction is reduced upon insulin stimulation, but this dissociation by insulin is impaired in AD lymphocytes. Although simufilam did not reduce the high levels of FLNA—IRβ, it markedly improved insulin’s ability to un-link FLNA from IRβ, potentially allowing IRβ to recruit IRS-1 to initiate signaling. Suggesting another possible mechanism, simufilam oral treatment restored FLNA’s normal linkage to the mTOR suppressor PTEN in AD lymphocytes. We hypothesize that the effects of simufilam on multiple disease-associated signaling pathways are mediated by reversing an altered conformation of FLNA. We further hypothesize that an altered conformation in AD leads to the hyperphosphorylation of FLNA at S^2152^ in AD lymphocytes, which was also reduced by simufilam. Implying translation to brain, *ex vivo* incubation with simufilam significantly reduced the high levels of pS^2152^FLNA in postmortem AD and amnestic MCI brain tissue.

Overactive mTOR and its resistance to insulin stimulation are considered pathological features of AD that represent insulin resistance independent of type-2 diabetes (Oddo, 2012; Norambuena et al., 2017), along with impaired insulin receptor function in AD postmortem brain (Talbot et al., 2012). Simufilam’s reduction of overactive mTOR and improvement to mTOR stimulation by insulin, detected across five mTORC1 and mTORC2 signaling components, is another mechanistic benefit of this therapeutic drug candidate. For mTORC1 and mTORC2 signaling, the elevated basal levels in AD lymphocytes prior to simufilam treatment were comparable to insulin-stimulated levels in healthy controls, leaving little room for further stimulation by insulin. The elevated basal activity of mTORC1’s downstream kinase p70S6K, however, was markedly below the insulin-stimulated level of healthy control lymphocytes, and its weak stimulation by insulin did not approach that of healthy controls. Simufilam both suppressed p70S6K basal levels and improved its stimulation by insulin, in fact, to a level higher than insulin-stimulated healthy controls. Similar patterns were shown for pT^1135^Rictor and Akt1, both phosphorylated by mTORC2. These findings show that the effect of simufilam is not just a suppression of overactive basal levels but also includes a lessening of mTOR’s insulin resistance. The effect of simufilam to reduce overactivation of both mTORC1 and mTORC2 signaling molecules and improve their stimulation by insulin suggests that simufilam may lessen mTOR’s contribution to AD pathology. In addition to improving insulin resistance of these mTOR parameters, simufilam has improved insulin receptor signaling both in postmortem AD brain and in AD mouse models, although not to normal levels (Wang et al., 2012; Wang et al., 2017).

The differences in mTOR activity induced by simufilam or insulin were not due to changes in mTOR association with either Raptor in mTORC1 or Rictor in mTORC2, because neither insulin nor simufilam treatment affected the levels of Raptor/Rictor association with mTOR. However, it is possible that the reduced Raptor (but not Rictor) association with mTOR in healthy control versus AD lymphocytes might indicate that different downstream signaling pathways for mTORC1 are prominent in the diseased versus normal lymphocytes, because differential binding of Raptor/Rictor to mTORC1/2 can affect substrate specificity for mTOR (Hay and Sonenberg, 2004; Su and Jacinto, 2011).

The absence of significant differences in mTOR parameters in lymphocytes of healthy control subjects in different age ranges, allowing for their combination into one control group, was unexpected, despite their small size (*n* = 6). However, the role of mTOR in aging has been demonstrated only in flies, worms and mice without substantial variations in diet, exercise, epigenetics and other factors (Johnson et al., 2013). The within-group variability in each age range of healthy control subjects suggests that besides disease, other factors may more strongly influence mTOR signaling than age itself.

The higher levels of FLNA linkage to IRβ of the insulin receptor in AD versus healthy control lymphocytes and the impaired response to insulin in reducing that FLNA—IRβ linkage in AD lymphocytes may also reflect insulin receptor dysfunction, as occurs in AD brain (Talbot et al., 2012). A plausible explanation for the dissociation of IRβ from FLNA upon insulin stimulation is that it may free IRβ to initiate signaling by linking to IRS-1, the signaling adaptor molecule that integrates downstream insulin receptor signaling cascades (Shaw, 2011). In support of this possibility, our unpublished data show minimal linkage of FLNA with IRS-1 in postmortem control brain. Interestingly, in A7 cells that overexpress FLNA, blocking the FLNA—IR linkage by ectopic expression of a C-terminal FLNA fragment blocked the MAPK cascade of the insulin receptor (He et al., 2003). In contrast to lymphocytes, there were no differences in FLNA—IRβ linkage in postmortem brain tissue between control, amnestic MCI, non-amnestic MCI and AD, nor was this linkage affected by exogenous Aβ_42_. The fact that FLNA—IRβ levels are comparable in control and AD brain but strikingly different in AD and control lymphocytes illustrates that this protein interaction and its disease influence varies by cell type or tissue, as is the case with mTORC2 (Knudsen et al., 2020). In healthy control but not AD lymphocytes, insulin reduced this FLNA—IRβ linkage, and simufilam treatment improved insulin’s ability to dissociate FLNA from IRβ in AD lymphocytes. The improved insulin-mediated FLNA—IRβ dissociation in AD lymphocytes by simufilam echoes simufilam’s improvement in IR signaling in postmortem AD brain and in AD mouse models (Wang et al., 2012; Wang et al., 2017). Together, the restored insulin-stimulated FLNA—IRβ dissociation and the restored responsiveness of mTOR to insulin show a coordinated improvement in insulin sensitivity in AD patient lymphocytes following simufilam.

The beneficial effects on mTOR signaling may be related to simufilam’s mechanism of action of reversing an altered conformation of FLNA that occurs in AD brain, implied by a shift in isoelectric focusing point (Wang et al., 2017; Wang et al., 2020). We previously demonstrated that FLNA in AD brain and lymphocytes aberrantly links to α7AChR and to TLR4, and these aberrant protein interactions (and resulting pathologies) are disrupted by simufilam and induced by Aβ_42_ (Wang et al., 2012; Wang et al., 2017; Wang et al., 2020). Because the shift in isoelectric focusing point is unaffected by complete dephosphorylation, an altered conformation is the most likely explanation for this shift (Wang et al., 2017). FLNA’s hyperphosphorylation at S^2152^ in AD brain and lymphocytes may be a consequence of the altered shape, especially as it was reduced by simufilam (*ex vivo* incubation of postmortem brain tissue or oral administration for lymphocytes). The fact that pS^2152^FLNA levels are elevated by incubation with exogenous Aβ_42_ indicates that soluble Aβ_42_ in AD brain or lymphocytes (Meng et al., 2019) can activate kinase(s) such as mTOR (Shaw, 2011) to phosphorylate FLNA. Simufilam facilitates dephosphorylation of pS^2152^FLNA and prevents further phosphorylation, perhaps by restoring FLNA’s native shape.

The reduction in pS^2152^FLNA by simufilam in lymphocytes following oral treatment and in postmortem brain tissue incubated *ex vivo* suggests that treatment effects in lymphocytes might mirror those in brains of AD subjects, but the significance of pS^2152^FLNA is not fully clear. pS^2152^FLNA has been implicated in FLNA—integrin interactions and their role in focal adhesion formation and cell migration of cancer cells (Sato et al., 2016). Phosphorylation of FLNA at S^2152^ was shown to occur by mTOR (Sato et al., 2016) and by IGF-1 in cancer cells (Ravid et al., 2008). Upon T cell receptor activation, the kinase Ndr2 phosphorylates FLNA at S^2152^, causing FLNA to dissociate from the inactive conformation of an integrin so that other proteins can stabilize this integrin’s active conformation (Waldt et al., 2018). pS^2152^FLNA has also been shown to block the anti-tumoral signaling of the somatostatin subtype 2 receptor, thereby exacerbating pituitary cancer (Peverelli et al., 2018). Importantly, simufilam reduced pS^2152^FLNA in human pituitary tumor cells and enhanced somatostatin subtype 2 receptor signaling (Marra et al., 2023). Elevated pS^2152^FLNA is also associated with poor prognosis and high-metastatic liver cancer (Xing et al., 2019). Overall, elevated pS^2152^FLNA appears to be pathological, and we propose that it is related to an altered conformation and altered protein interactions of FLNA in AD.

The reduced PTEN—FLNA interaction in AD lymphocytes may be another consequence of altered FLNA. We hypothesize that the disruption of the FLNA—PTEN interaction in AD lymphocytes, and presumably also in brain, may impair PTEN’s negative regulation of the mTOR pathway, contributing to the overactive mTOR signaling in AD. Simufilam’s reduction of pS^2152^FLNA and improved FLNA—PTEN linkage in lymphocytes of AD subjects supports this theory. AD-related changes to FLNA, both in conformation and phosphorylation (at S^2152^ and potentially other sites) may impact multiple protein interactions of FLNA, including the reduced interaction with PTEN shown here in AD brain and lymphocytes. Their reversal by simufilam, which shifts the majority of FLNA in AD back to its native state (Wang et al., 2017; Wang et al., 2020), also suggests the reduced PTEN tethering to FLNA and FLNA’s hyperphosphorylation in AD are consequences of an altered conformation of FLNA. However, whether the restored PTEN tethering directly dephosphorylates pS^2152^FLNA requires further research.

With important implications for AD, mTOR signaling is known to regulate synaptic plasticity and long-term potentiation (LTP), affecting memory formation (Lipton and Sahin, 2014; Bockaert and Marin, 2015). Long-term fear memory mediated by medial prefrontal cortex in rat has been shown to require mTOR activation, and LTP induction by high-frequency stimulation activates mTOR and p70S6K (Sui et al., 2008). Additionally, direct administration of PI3K inhibitors together with rapamycin into the medial prefrontal cortex suppressed both induced LTP and long-term fear memory (Sui et al., 2008). These findings imply that improved mTOR signaling may benefit synaptic plasticity and memory in AD patients.

## Conclusion

The suppression of overactive mTOR signaling and its improved responsiveness to insulin represents a mechanistic benefit of simufilam beyond the disruption of pathogenic signaling pathways of soluble amyloid. These improvements in mTOR signaling may similarly result from reversing an altered conformation of FLNA, here allowing insulin to dissociate FLNA’s linkage to the insulin receptor and initiate signaling. Further, restoring PTEN tethering to FLNA may allow PTEN to regulate mTOR. Although this work relied on lymphocytes from AD subjects before and after treatment with simufilam compared to healthy control lymphocytes, these treatment effects shown in lymphocytes likely translate to mTOR in brain and other tissues, as evidenced by simufilam’s reduction of FLNA hyperphosphorylation in postmortem human AD brain. Because mTOR contributes to age-related cellular changes, simufilam’s suppression of mTOR overactivation concurrent with improved insulin sensitivity may slow certain aging processes and attenuate this pathological feature of AD, potentially benefiting brain function and memory in AD and in aging.

## Data Availability

The datasets presented in this article are not readily available because the subject-level research data will not be shared. Requests to access the datasets should be directed to Eric Schoen, eschoen@cassavasciences.com.
