# Expression of concern: Simufilam suppresses overactive mTOR and restores its sensitivity to insulin in Alzheimer’s disease patient lymphocytes

**DOI:** 10.3389/fragi.2024.1483030

**Published:** 2024-08-22

**Authors:** Frontiers Editorial Office

With this notice, Frontiers states its awareness of concerns regarding the images for “Simufilam suppresses overactive mTOR and restores its sensitivity to insulin in Alzheimer’s disease patient lymphocytes” published on the 29th of June 2023. An investigation is currently being conducted in accordance with COPE guidelines. This notice will be updated as soon as the investigation is complete.

**UPDATE:** Following concerns regarding the images in “Simufilam Suppresses Overactive mTOR and Restores Its Sensitivity to Insulin in Alzheimer's Disease Patient Lymphocytes,” published on 29 June 2023, Frontiers launched an investigation in accordance with COPE guidelines. As part of this process, the authors provided the original Western blot images for the study. After careful examination, Frontiers found no evidence of manipulation of the Western blot data. Frontiers will review any additional findings from the institutional investigation as they become available.

